# Sedation Methods in Paediatric Auditory Electrophysiologic Testing: A Narrative Review

**DOI:** 10.3390/audiolres15040082

**Published:** 2025-07-04

**Authors:** Violeta Necula, Maria Eugenia Domuta, Raluca Olariu, Madalina Gabriela Georgescu, Ioan Florin Marchis, Mirela Cristina Stamate, Cristina Maria Blebea, Maximilian George Dindelegan, Alma Aurelia Maniu, Sever Septimiu Pop

**Affiliations:** 1 Otorhinolaryngology Department, “Iuliu Hatieganu” University of Medicine and Pharmacy Cluj-Napoca, 400012 Cluj-Napoca, Romania; 2 Emergency County Clinical Hospital Cluj-Napoca, 400006 Cluj-Napoca, Romania; 3 Otorhinolaryngology Department, University of Oradea, 410037 Oradea, Romania; 4 Otorhinolaryngology Department, “Grigore T. Popa” University of Medicine and Pharmacy, 700115 Iasi, Romania; 5 Audiology Department, Clinical Rehabilitation Hospital Iasi, 700661 Iasi, Romania; 6 Otorhinolaryngology Department, “Carol Davila” University of Medicine and Pharmacy, 050474 Bucharest, Romania; 7 Otorhinolaryngology Department, Institute of Oncology “Prof. Dr. Ion Chiricuta”, 400015 Cluj-Napoca, Romania

**Keywords:** ABR, ASSR, electrophysiologic auditory tests, sedation, melatonin, chloral hydrate, dexmedetomidine

## Abstract

Implementing neonatal hearing screening has significantly reduced the age at which hearing impairments are detected in children. Nevertheless, objective electrophysiological assessments, such as auditory brainstem response (ABR) or auditory steady-state response (ASSR) testing, are often necessary for children older than six months. These evaluations should be conducted while the child is asleep to obtain accurate and interpretable results, as movement and muscle activity can introduce artifacts that compromise the quality of the recordings. In this narrative review, we evaluate sedation strategies in paediatric procedures, focusing on the efficacy, safety, and practicality of agents/routes for inducing sleep during ABR/ASSR testing. Sedation choices should prioritise patient safety, clinical context, and result reliability and be tailored to the individual’s age, health status, and procedural needs.

## 1. Introduction

Hearing screening in newborns has significantly lowered the average age for hearing loss detection. Young children who fail hearing screening or older children who require audiological evaluation or re-evaluation can be referred to specialised diagnostic centres. Early testing means timely diagnosis and intervention, leading to better results.

The audiological tests used to assess children’s hearing include objective and behavioural methods. While behavioural tests require certain responses from the child, dependent on the child’s age, objective audiological measures record various responses independently of the child’s participation. These techniques include electrophysiological and electroacoustic methods, where some, such as otoacoustic emissions and impedancemetry, require a quiet, non-crying child, while others, such as auditory evoked potentials (ABR) and auditory steady-state response (ASSR), require the child to sleep [1].

In electrophysiological tests, the small voltage changes generated by the nerve structures that make up the auditory pathway in response to an auditory stimulus are collected with surface electrodes. The analysis of these responses allows for the assessment of the transmission and processing of auditory information. ABR testing can be used to objectively assess pathways from the peripheral to the central auditory system and has a major impact on the detection of hearing disorders in children [2]. The advantage of the ABR is that it can be recorded at any age, regardless of the attention or sleep state. Meanwhile, ASSR testing measures the response to modulated or repetitive acoustic stimuli and reflects the activity of the brainstem and auditory cortex; depending on the modulation rate, the brainstem responds at higher rates than the cortex. The ASSR test can be conducted at any age, and its testing can be performed independently of the sleep state or anaesthesia [3]. However, even though these tests can be performed while the child is awake, the relatively long duration of the procedure requires testing children in their sleep to obtain interpretable tracings.

ABR and ASSR are far-field recordings with low amplitude that are difficult to select from mixed EEG signals. Recording appropriate ABR waves requires amplification and noise reduction to maximise the signal-to-noise ratio, alongside fitting averaging and artefact rejection strategies. Sleep provides reduced EEG activity, improving the signal-to-noise ratio and allowing for the easier selection of ABR waves with reduced amplitude.

Muscle activity also has a negative effect on ABR recordings. Maruthy et al. (2015) showed that blinking and the contraction of muscles of the face, jaw, neck, lips, and cheek can interfere with ABR recordings [4]. Movements of the body, especially of the head or mandibular movements, produce myogenic potentials or electrical artifacts [5]. As such, the patient should be as quiet and relaxed as possible and neither talk nor move the head.

Ambient environmental noise can influence the recording of ABR traces and hinder the interpretation of the results due to difficulties in separating signal from noise [6]. Noise can also elongate the latency and reduce the amplitude of ABR waves [7,8]. Although Richmond et al. [9] and Dzulkarnain et al. [10] claim that ambient acoustic noise of up to 60 dBA does not significantly influence ABR waves and latency in adults, quieter environments are still recommended, particularly in children, to ensure optimal recordings.

Electromagnetic interference from electrical equipment in operating rooms or testing environments can significantly compromise the quality of ABR recordings. It is essential to implement strategies that minimise such interference to ensure accurate and reliable results. The proper grounding of equipment and the elimination of significant sources of electrical noise can reduce electromagnetic interference during ABR testing, leading to more accurate assessments of auditory function [11].

ASSR results are also influenced by ambient noise, electromagnetic interferences, or muscular activity. The higher the noise level, the lower the amplitude of the waves, and the greater the difficulty in recognising the response [12]. It is, therefore, advisable to perform these tests on a quiet patient, preferably asleep, for the duration of the procedure.

Most children under the age of 6 months can be tested during natural sleep. However, at older ages, sedation may be necessary to perform the auditory test. Several studies have addressed the use of different drugs, comparing factors including administration routes (oral, intranasal, or intravenous) and outpatient, inpatient, or operating room settings. However, no consensus has been achieved, and no guidelines have been published on appropriate means and conditions of sedation [13]. Hence, we conducted a literature review examining sedation protocols for children undergoing electrophysiological hearing tests (ABR and ASSR). Subsequently, we expanded the analysis to include studies where the same sedative agents were used for procedural sedation in non-auditory clinical contexts (e.g., MRI, CT, dental procedures, and endoscopy). This dual approach was adopted to establish comparative insights into dosage ranges, onset times, and duration of action across applications.

## 2. Testing Conditions

### 2.1. Natural Sleep Testing

Younger children can most often be tested in natural sleep. In a study group of children of 4 months on average, Jenssen et al. (2010) reported a natural sleep duration of 48.8 min, with 20% having a shorter duration of up to 33.1 min. One conclusion of the study was that the testing duration of around 60 min exceeded the average child’s natural sleep duration, except in normal hearing cases, where the duration was shorter [14].

Natural sleep testing relies heavily on cooperation between the testing centre and the child’s family. The likelihood of the child falling asleep for the duration of the test can be increased through sleep deprivation prior to the session, diaper changing, and the feeding of the child just before the procedure. Behavioural and cognitive therapy could be considered for sleep induction. The preparation of the skin, the application of the electrodes, and the insertion of earphones can often be performed before the child falls asleep [15].

### 2.2. Drug-Induced Sleep Testing

Sedation is a reduction in consciousness following the administration of certain drugs. It is also usually associated with reduced anxiety and can induce retrograde amnesia. The muscular relaxation caused by these drugs can cause breathing disturbances and cardiovascular reflexes such as bradycardia or hypotension. A wide range of drugs are used precisely because there is no ideal example that provides the necessary total sedation, avoids the risk of severe complications such as cardiorespiratory depression, and allows for rapid awakening. The route of administration can be oral, intranasal, intrarectal, intravenous, or via inhalation; depending on the case, the latter can require respiratory support with a laryngeal mask or orotracheal intubation in an operating theatre. The advantage of oral or intranasal sedation is the possibility of administering it outside of the operating theatre, as it requires less complex monitoring than respiratory support. Patients classified as ASA I or II are generally considered suitable candidates for minimal, moderate, or deep sedation. However, children with severe systemic conditions (classified as ASA III–V) and patients with special healthcare needs, anatomical airway abnormalities, or moderate-to-severe tonsillar hypertrophy require individualised assessment and monitoring by an anaesthesiologist during sedation [16,17]. Like any medical act, sedation has its risks, including breathing disorders—such as airway obstruction or hypoventilation—aspiration, and cardiovascular disorders [18]. Obesity increases these risks and requires special attention, especially when associated with sleep apnoea [19]. The medical staff involved must be able to recognise potential complications, the most significant of which include airway compromise, depressed respiration, hypoventilation, laryngospasm, hypoxemia, and apnoea. Cardiac complications are usually secondary to untreated respiratory disturbances. Other possible complications may include seizures, vomiting, and allergic reactions [17].

Pre-sedation assessment of the child and physical examination are mandatory to identify possible risk factors so that the procedure can be performed as safely as possible. Fasting recommendations prior to sedation are as follows: 2 h for clear liquids, 4 h for breast milk, 6 h for nonhuman milk or infant formula, and 6 h for light solid foods. During oral or intranasal paediatric procedural sedation, the continuous monitoring of oxygen saturation, heart rate, and ventilation is strongly recommended, accompanied by a regular assessment of blood pressure and respiratory rate. Capnography is recommended for nearly all children undergoing deep sedation due to the heightened risk of airway and ventilation compromise. The presence of appropriately trained personnel and readily available resuscitation equipment is critical to ensuring prompt emergency intervention, should complication arise. Monitoring should continue until the child is fully alert and stable [17].

#### 2.2.1. Oral, Intranasal, or Rectal Administration

Paediatric procedural sedation (PPS) is a drug-induced depression of consciousness that helps patients tolerate unpleasant or prolonged medical procedures by reducing anxiety, discomfort, and pain. The American Society of Anesthesiologists (ASA) classifies most procedural sedation as moderate sedation/analgesia, though deep sedation/analgesia may be required for highly painful interventions. Standard guidelines inadequately address the challenges of managing uncooperative paediatric patients, particularly in non-painful electrophysiological testing (e.g., auditory brainstem response), where procedural success is dependent on the achieving complete immobility [20].

During moderate sedation, patients are maintained at a sedation level at which they are responsive to verbal/tactile stimuli, maintain spontaneous ventilation, and require no airway interventions. Cardiovascular stability is typically preserved [21,22]. Paediatric procedural sedation protocols often integrate sedation, analgesia, and dissociation, depending on the nature of the procedure. In the case of electrophysiological tests, which are not painful, the primary goal is to provide adequate sedation to ensure that the child remains asleep throughout the test. Medications used for oral or intranasal sedation tend to have a slower onset and less predictable effects and may occasionally fail to achieve the desired level of sedation [23].

Therefore, in healthy paediatric patients undergoing minimal sedation for non-painful procedures such as audiometric testing, intranasal (IN) or oral midazolam or IN dexmedetomidine is preferred over short-acting barbiturates. These agents can be safely administered without intravenous access by non-anaesthesiologist professionals (e.g., ENT specialists) provided they have received appropriate training, can ensure continuous patient monitoring, and have immediate access to emergency backup support if needed [24,25].

- Melatonin is a hormone (N-acetyl-5-methoxytryptamine) naturally produced by the pineal gland that plays a key role in controlling the sleep–wake cycle. Exogenous melatonin has been shown to reduce sleep onset latency and increase both the efficiency and duration of sleep [26]. No significant side effects have been reported in the literature in either adults or children, and its use does not require close medical monitoring [27]. The dosage of melatonin administered varies across studies; Andersen et al. (2014) reported values ranging from 3 to 10 mg in a review [28], while in a separate systematic review, Behrman et al. (2020) noted dosages ranging from 0.25 mg in children under 3 months to 20 mg in children over 6 years [29]. The effectiveness of melatonin is highly variable. Behrman et al. (2020) reported a success rate between 65% and 86.7%, with more success in children under 1 year of age and lower rates in those over 3 years [29]. In a study by Hajjij et al. (2020), melatonin was administered to 247 children with a mean age of 2 years and 4 months. They found that 75.7% of the children completed full testing, while 24.27% experienced interrupted sleep, and most required additional doses [30]. Casteil et al. (2017) administered 5 or 10 mg of melatonin to 29 children aged between 1 and 6 years, achieving sufficient sleep for complete testing in 59% of the children, with a failure rate of 27% [31]. Meanwhile, Schmidt et al. (2007) reported failure rates of only 4% in children under the age of 1 year and 25% in children older than 3 years [27]. In a group of 33 children aged between 5 months and 4 years (with a mean age of 2 years and 8 months), Chaouki et al. (2020) reported a failure rate of 27.3%. The onset of melatonin’s effect was reported between 15 and 55 min, with a mean onset time of 30.39 min. Additionally, 48.5% of the children required an additional dose of melatonin to achieve the desired effect [32].
- Chloral hydrate is a non-opioid, non-benzodiazepine sedative and hypnotic drug. It is commonly used in paediatric audiology, as well as in neurological, imaging, and dental investigations or treatment. Although considered effective and safe in adequate doses, its use is banned in some countries because of the potentially severe adverse effects at higher doses; possible carcinogenic effects have also been observed in guinea pigs but have not yet been confirmed in humans [33,34]. Despite these concerns, chloral hydrate is considered safe and effective for children undergoing painless diagnostic procedures [35]. Valenzuela et al., in a study in 635 children, used an average dose of 52 mg/kg and achieved a 95.9% success rate. Side effects were reported in 19.2% of patients, including 3.4% who had severe complications such as apnoea or bradycardia; furthermore, 6.2% had minor complications, such as vomiting, hypoxemia, prolonged sedation, and tachypnoea, and 5% suffered agitation [36]. Vomiting is the most common adverse effect. Avlonitou et al. (2011) recorded an incidence rate of 11.4% [35], similar to the 11.5% reported by Necula et al. (2019) [37], while Liu et al. (2024) reported a much lower incidence of 0.25% [38].

Agitation was the second most common adverse effect, with an incidence rate of 5% being reported by Valenzuela et al. [36], 8% by Avlonitou et al. [35], and 3.1% by Necula et al. [37].

In a large study conducted by Xiangling Zhang et al. in a group of 6106 children, a failure rate of 3.11% was reported for a dose of 30 mg/kg, with a higher rate of 4.31% in the 0.5–3-year age group [39]. A meta-analysis published by Liu et al. (2024) included 23 studies on the use of chloral hydrate for paediatric sedation. The pooled sedation failure rate was 10.0%, and the overall incidence of adverse reactions was 10.32% [38].

A frequently mentioned negative aspect is the bitter and unpleasant taste of choral hydrate. It is also a gastric irritant, often causing vomiting, especially when administered in the large volumes needed for children with higher body weight [23].

Chloral hydrate can also be administered rectally, with a lower incidence rate of adverse events than the oral route. According to a systematic review conducted by Chen et al., the dose used in various studies ranged from 20 to 80 mg/kg [40]. Nie et al. reported a mean sleep onset time of 16.41 min, with onset occurring faster in children under 12 months and slower in those over 12 months. The mean sleep duration was 71.59 ± 20.60 min. In their study, a decrease in heart rate, respiratory rate, and oxygen saturation was observed; however, all values remained within normal limits [41].

- Triclofos is the active metabolite of chloral hydrate, specifically the sodium monophosphate salt of trichlorethanol [42]. It is better tolerated than chloral hydrate, as it causes less gastric irritation, but has a longer onset time [23]. The typical dose of triclofos is 50 mg/kg, with the option to administer an additional dose if sleep does not occur within 30 min. Jain et al. administered triclofos to a group of 160 children aged 14 to 36 months; 17.5% required an additional dose. The median sleep latency was 30 min, and the median sleep duration was 90 min. The reported side effects included dizziness, irritability, and vomiting, with no severe complications or respiratory disturbances. The success rate was 93.1% [43].

Studies have shown that triclofos can be safely used in children with congenital heart disease or neurological disorders. It is widely used in India but has been banned in the United States since the 2000s.

- Hydroxyzine dihydrochloride (Atarax) is the hydrochloride salt of hydroxyzine, a first-generation antihistamine and H1 receptor agonist with antiallergic, antispasmodic, sedative, antiemetic, and anxiolytic properties. The recommended paediatric dose for children weighing less than 40 kg is 2 mg/kg. The onset of action occurs in 15 to 60 min, with a duration of effect of approximately 4 to 6 h [44]. Reported side effects include prolonged QT/QTc intervals on the echocardiogram, and the drug should be used with caution in patients with porphyria or pre-existing QT prolongation [42]. Overdose can lead to hyper-sedation, seizures, stupor, nausea, and vomiting. In such cases, gastric lavage, symptomatic management, and supportive care are indicated [45]. It is more efficient when used in combination with other agents, such as nitrous oxide [46].
- Midazolam is a short-acting benzodiazepine widely used in paediatric hospital practice. It is used for its anxiolytic, sedative, anterograde amnestic, and muscle relaxant properties and can be administered through various routes—intravenous, oral, buccal, intranasal, or rectal—each with specific advantages and limitations [47,48]. The oral bioavailability of midazolam in children has been reported to range between 15% [49] and 36% [50], while in adults, the values range from 31% to 72% [51]. The lower bioavailability in children suggests that higher doses are required than in adults. According to Higuchi et al. [52], a dose of 0.32 ± 0.10 mg/kg is appropriate for achieving sedation levels classified from drowsy, sleepy, and lethargic to asleep, corresponding to levels 2 and 3 on the sedation scoring system developed by Yuen et al. [53]. A deeper sedation level (level 4) is typically achieved only at higher doses. Manso et al. suggested that the optimal dose in children is 0.5/kg [54]. Adverse effects reported in the literature include paradoxical reactions, nausea, vomiting, and respiratory events, most commonly observed at doses exceeding 0.5 mg/kg [55]. A drawback of oral administration is the unpleasant taste, which is difficult to mask even with flavourings, often resulting in spitting or regurgitation by children [56]. The intranasal route offers the advantage of faster absorption into systemic circulation—resulting in a quicker onset, a shorter duration of action, and faster recovery—due to its higher bioavailability compared with the oral route. It also confers anterograde amnesia [57]. However, intranasal administration is often poorly tolerated by children due to the tingling or burning sensation, as the concentrated solution has an irritant effect on the nasal mucosa. The recommended dose is 0.5 mg/kg administered intranasally. According to Stephen et al., the onset of action occurs in 5 to 10 min, with the effect lasting up to 108 min. However, the success rate in monotherapy is relatively low, reaching only 51% [58]. Side effects may include nausea, vomiting, and cognitive and respiratory problems [59,60]. Midazolam, whether administered orally or intranasally, is frequently combined with intranasal dexmedetomidine to enhance sedative efficacy.
- Dexmedetomidine (DEX) is a relatively new anxiolytic, sedative, hypnotic, and analgesic drug that acts as a selective agonist of alpha-2 adrenergic receptors in the central nervous system [61]. One of its major advantages appears to be its stronger safety profile, including a lack of respiratory depression [62]. The drug is absorbed through the nasal mucosa, which allows for intranasal administration as an alternative to the intravenous route. This is particularly beneficial in non-cooperative paediatric patients, as it avoids the pain and stress associated with intravenous catheter placement [63].

The onset time of sleep induction following intranasal DEX varies between 10 and 60 min, with an average of 22 min [62]. Reynolds et al. reported a success rate of 89% following a single intranasal dose, with a mean onset time of 25 min [64]. While the success rate of DEX is comparable to that of chloral hydrate, the longer sleep onset time, ranging from 20 to 40 min, is often considered a disadvantage, as it can be a limitation in a busy clinical environment [65].

In a 2022 review, Marra et al. analysed six studies on intranasal DEX from 2015 to 2021. The doses ranged from 2 to 4 µg/kg, with 3 µg/kg being the most common. The reported success rates varied from 82.5% [66] to 100% [62]. Gupta et al. reported that 14% of children (out of a cohort of 203) required dose supplementation, 6% needed oxygen support, and the failure rate was 2% [67]. Giordano et al. reported a success rate of 96.6% in a group of 59 children (mean age 3.0 ± 1.6 years) following an initial dose of 2.5 µg/kg, with an additional 1 µg/kg being administered at 30 min if sedation was incomplete. The mean onset of sedation was 32.4 ± 18.3 min. In their cohort, 48.3% experienced hypotension and 53.5% bradycardia, although medical intervention was not required. In this study, the success rate was 96.6% [68]. Tug et al. also reported mild bradycardia and hypotension without necessitating treatment [69]. In a larger cohort of 578 children, Tsze et al. supplemented sedation with oral or intranasal midazolam in 39.3% of cases, achieving complete procedural success in 91.3% of children. The reported adverse effects included bradycardia in 1.9% of patients and oxygen desaturation in 0.9%, with no severe complications [70].

In a meta-analysis, Tervonen et al. concluded that intranasal DEX has a comparable success rate to chloral hydrate but with a lower incidence rate of nausea and vomiting. Moreover, DEX demonstrated a higher success rate than midazolam [71]. Li et al. found that the combination of intranasal DEX (3 µg/kg) and intranasal or buccal midazolam (0.1–0.2 mg/kg) produced a higher success rate (97.5%) than DEX alone [66].

- Pentobarbital has been more widely used in procedural sedation, particularly via intravenous administration. Common side effects include hypotension, respiratory disturbances, prolonged recovery time, and paradoxical reactions [72]. Oral administration has a high reported success rate, 82% in the study conducted by Anderson et al. (2008), with a low rate of complications aside from a longer sleeping time [73]. The oral dose of pentobarbital (50 mg/mL) reported by some authors is 4 mg/kg, with an additional 2 mg/kg to be administered as needed, up to a maximum dose of 8 mg/kg [72]. Pentobarbital with or without alimemazine was used by François et al. (2011) in a group of 180 children aged between 2 and 5 years. They administered intrarectal pentobarbital or intrarectal pentobarbital and oral alimemazine with a success rate of 89.8%. The mean sleep onset time was 64 ± 40 min [74]. Intrarectal pentobarbital at a dose of 5 mg/kg was also used by Baculard et al. (2007) in a group of 68 children under the age of 8 years. The average time to sleep onset was 36.1 min, with a success rate of 89.7%. Adverse effects were reported in 15.9% of cases [75].
- Clonidine, originally developed as an antihypertensive agent, has gained increasing recognition in recent years for its sedative, anxiolytic, and analgesic properties [76]. Notably, clonidine does not cause respiratory depression and could be a suitable option for children with sleep apnoea or other respiratory disorders. In contrast to midazolam, clonidine does not impair cognition and memory [77]. Jatti et al. demonstrated that clonidine provides effective sedation and can be reliably used as a premedication agent in paediatric patients [78]. Clonidine can be administered orally (4–5 µg/kg), intranasally (2–4 µg/kg; but this route may cause nasal irritation or burning), or rectally (2.5–5 µg/kg) and is often co-administered with atropine (40 µg/kg). It may also be administered via intramuscular injection (2 µg/kg) or intravenously (1–2 µg/kg as a bolus followed by continuous infusion at 0.18–3.16 µg/kg/hour). It can be used as a sole agent or in combination with midazolam (50 µg/kg) [79].

The advantages and disadvantages of the various administration routes, as well as their indications, can be found in Table 1.

#### 2.2.2. Deep Sedation and General Anaesthesia: Intravenous and/or Inhalation Administration with or Without Respiratory Support

Deep sedation is a drug-induced depression of consciousness, during which patients cannot be easily aroused but may respond purposefully to repeated or painful stimulation. In contrast, general anaesthesia is a drug-induced loss of consciousness, during which patients are not arousable, even by painful stimulation. The ability to independently maintain ventilation is often impaired; patients may, therefore, require assistance in maintaining a patent airway. Monitored anaesthesia care (MAC) refers to a specific anaesthesia service performed by a qualified anaesthesiologist during a diagnostic or therapeutic procedure, encompassing the full range of sedation levels, up to and including the transition to general anaesthesia [80].

Deep sedation and general anaesthesia require the presence of qualified personnel who are capable of administering the necessary pharmacological agents and promptly intervening to secure the airway in case of complications [81]. The most commonly used combination includes benzodiazepines and opioids, although other drug combinations may also be employed. The route of administration is usually intravenous or inhalational. The main advantages of this method lie in the rapid onset of sedation and the ability for the process to be closely monitored and adjusted by an anaesthesiologist, who is trained to promptly identify and manage adverse effects or complications [82]. Deep sedation or general anaesthesia administered by anaesthesiologists is generally recommended in children with comorbidities that may be exacerbated by sedation (ASA physical status III or IV), as well as those with special needs, anatomical airway abnormalities, or moderate-to-severe adenotonsillar hypertrophy. In such cases, procedures should be performed in a specialised facility equipped for general anaesthesia and advanced airway management, including endotracheal intubation or the use of a supraglottic airway device [17,25].

- Midazolam can be administered intravenously, initially at a higher dose of 2–2.5 mg, followed by supplementary doses of 1 mg every 2–5 min, depending on the effect. Its onset is rapid, typically occurring within 2–3 min [83].
- Fentanyl is a synthetic opioid, administered intravenously at an initial dose of 1–1.5 µg/kg, followed by a maintenance dose of 1 µg/kg every 3 min. The onset of action occurs in 1–2 min and lasts between 30 and 60 min [82].
- Ketamine can be administered intravenously at a dose of 1–3 µg/kg or intramuscularly at 5–10 µg/kg. Its onset of action is rapid, within 1 min, and the duration of effect ranges from 15 to 30 min, depending on the route of administration [84]. An advantage of ketamine is the maintenance of haemodynamic stability and spontaneous respiration, with only a mild bronchodilatory effect [85]. Common side effects include nausea, vomiting, hypersalivation, dizziness, diplopia, drowsiness, dysphoria, confusion, and hallucinations [86]. Respiratory complications such as laryngospasm and apnoea have also been reported [87].
- Propofol is an intravenously administered sedative–hypnotic drug. The recommended dose for children is 2–3 mg/kg, which can be repeated as needed. The onset of action occurs in 15–30 s and lasts between 1 and 3 min [88]. Recovery is rapid, and the medication is generally well tolerated [89]. The risk of apnoea and desaturation is the highest during induction [90]. Levit et al. (2018) administered propofol for ABR testing in a group of 126 children of over 24 months of age, using an initial bolus dose of 0.8 mg/kg followed by continuous infusion at a rate of 0.1 mg/kg/min [91].
- DEX, when administered intravenously at a dose of 1 µg/kg, has a rapid onset of action, inducing sleep within 3–5 min and lasting approximately 15 min, with the advantage of not causing respiratory depression [92].
- Nitrous oxide (N_2_O) is an analgesic and anxiolytic gas with rapid onset and quick recovery. It is administered mixed with oxygen with a face mask typically at a flow rate of 5–6 L/min [93]. Prolonged exposure to high levels of nitrous oxide (N_2_O) can lead to serious neurological damage, including neuropathy and even paralysis caused by cobalamin (vitamin B12) deficiency [94]. Other reported adverse effect are nausea, vomiting, dizziness, and confusion [95].
- Sevoflurane is administered with a face mask and does not require intubation. After induction, the maintenance dose can be reduced to a level that sustains the sleep state [96]. Various studies have shown that sevoflurane may favour false positive responses, resulting in ABR responses at higher intensities than those obtained through behavioural testing or with other drugs, such as propofol [97,98].
- The combination of propofol and ketamine is considered more effective than propofol alone, with fewer side effects such as bradycardia and hypotension. This combination helps minimise ketamine-induced vomiting and emergence reactions while also offsetting the hypotensive effects of propofol. The addition of low-dose ketamine reduces the required dose of propofol, thereby decreasing the risk of respiratory complications. The recommended dose is 1.5 mg/kg propofol with 0.5 mg/kg ketamine [99].
- The combination of intravenous ketamine (1 mg/kg) and midazolam (0.1 mg/kg) is an established rescue protocol for failed “safe sleep” sedation, especially in uncooperative paediatric patients undergoing procedures requiring rapid immobility. Midazolam helps mitigate ketamine-induced emergence reactions such as hallucinations. This combination has a rapid onset, typically within 30 s, and provides sedation lasting between 15 and 60 min without causing hypotension, bradycardia, or respiratory depression that would necessitate airway support or reversal agents [100].
- The combination of propofol and dexmedetomidine provides cardiovascular stability and early onset time without adverse effects such as airway obstruction, hypoxia, and spontaneous movement [101]. Dexmedetomidine mitigates propofol-induced hypotension through α_2_-adrenergic-mediated sympathetic inhibition, thereby stabilising heart rate and blood pressure. Additionally, it counteracts propofol-induced respiratory depression. Combined use reduces propofol requirements by 30–40% [101].
- Auditory testing under general anaesthesia with endotracheal intubation (EET) or a laryngeal mask airway (LMA) is recommended when the airway cannot be maintained with less invasive means. This is typically the case in children with multiple comorbidities, when there is a risk of aspiration, or in the presence of cardiovascular instability [102]. In such cases, testing should be performed in the operating room, in the presence of an anaesthesiologist team. Throughout the procedure, the anaesthesiologist monitors blood pressure, oxygen saturation, and heart rhythm. General anaesthesia involves a combination of drugs, such as midazolam for premedication and sevoflurane for induction, followed by propofol and fentanyl, with sevoflurane for maintenance [103]. The main disadvantage of this setting is the use of higher drug doses, which may prolong both induction and recovery times and increase the risk of side effects [23]. Additionally, higher doses of anaesthetic agents may result in longer ABR wave latency and reduced amplitudes, making interpretation more difficult, increasing the risk of false positives, and leading to the overestimation of the severity of hearing loss [98]. This effect has been demonstrated in several studies. Norrix et al. analysed the depressant effect of anaesthetic agents on brainstem neural activity in response to click stimuli and found prolonged I–III, II–V, and I–V latency values [104]. Similar findings have been reported elsewhere. Furthermore, interpretation is complicated in this context by background noise and electromagnetic interference from operating room equipment [103,105].

Reversal agents are used to promptly counteract the effects of sedatives and analgesics, particularly when excessive sedation leads to respiratory depression. Naloxone is the primary reversal agent for opioid-induced respiratory depression, while flumazenil is used to reverse the effects of benzodiazepines. For opioid overdose, naloxone is typically administered intravenously, with an initial dose ranging from 0.05 mg to 2.0 mg. If there is no response, the dose may be repeated every 2–3 min as needed, titrated to restore adequate spontaneous ventilation and respiratory function [106]. Flumazenil is administered intravenously for benzodiazepine reversal, starting with an initial dose of 0.2 mg intravenously given over 2 min. This dose can be repeated every minute with 0.2 mg increments until the desired level of consciousness is achieved, up to a maximum total dose of 1 mg [82].

**Figure 1:**
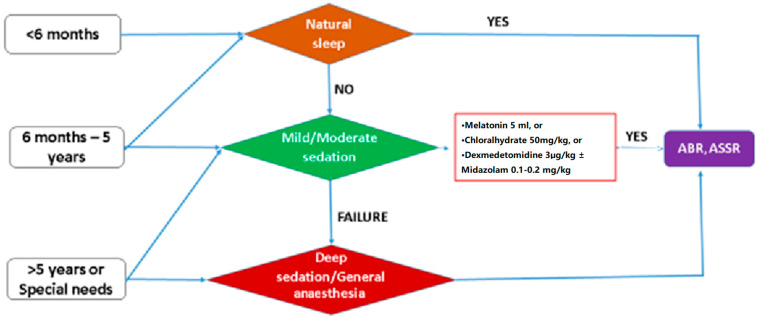
Clinical decision-making flowchart based on the child’s age and the most commonly used sedation drugs.

Considering the most extensively studied and effective pharmacological agents for sedation in paediatric auditory electrophysiological assessments, we developed a structured flow chart that guides substance selection based on the patient’s age. This approach integrates current evidence and clinical best practices to facilitate optimal sedation choices tailored to developmental considerations, thereby enhancing both the safety and efficacy of auditory testing in paediatric populations. (Figure 1)

A comprehensive overview of the medications used for paediatric sedation is provided, detailing the recommended doses, onset and duration of action, success rate, and potential adverse effects is presented in Table 2. A summary of age-based sedation strategies, including preferred agents and clinical considerations, is systematically outlined in Table 3.

## 3. Discussions

Hearing testing in children using electrophysiology is best performed while the child is asleep, as this provides the optimal conditions for obtaining interpretable results and ensuring an accurate diagnosis. Each method available for inducing sleep has its own advantages and limitations, and healthcare teams must carefully select the safest and most effective approach based on the specific needs of the child and the resources available.

Natural sleep offers the significant benefit of avoiding pharmacological side effects. However, it is frequently interrupted in children and may not last long enough for the completion of tests. Achieving natural sleep requires close cooperation from the child’s caregivers, including adherence to specific preparation protocols, something that may not always be feasible, particularly when families travel long distances to the clinic. While natural sleep can typically be induced more easily in infants below 6 months of age, it becomes increasingly challenging as the child grows older, often necessitating extended testing time.

Oral or intranasal sedation is a non-invasive option that does not require the presence of an anaesthesiologist and involves minimal monitoring. This makes it feasible outside the operating room. However, it requires personnel who are trained to recognise and manage potential side effects, are skilled in resuscitation, and can access intensive care support if necessary. The onset of sedation is slower and less predictable, and the success rate varies depending on the drug used. Chloral hydrate has historically demonstrated a high success rate, but its use is declining and is even banned in some countries. Intranasal DEX shows similarly high efficacy, with the added benefits of lower dosage requirements and fewer side effects such as vomiting. Although there is some risk associated with these sedatives, they allow for testing to be performed in more favourable acoustic and electromagnetic environments compared with the operating room and at a lower cost.

Deep sedation, facilitated by an anaesthesia team, provides more predictable and controlled sedation, with continuous monitoring and support for resuscitation if needed. This approach allows for a stable testing window and better control over the procedure’s duration. However, it must be carried out in an operating room, where the presence of medical equipment can increase acoustic and electromagnetic noise, potentially affecting the quality of the recordings. Although the risk of side effects exists, it is mitigated by the presence and expertise of the anaesthesia team. The disadvantages of this method include its invasiveness, the need for specialised personnel and equipment, and significantly higher costs.

This analysis has several methodological constraints. While focusing on sedation for electrophysiological testing in young children (e.g., ABR and ASSR), this review was expanded to include studies where sedatives were used for unrelated purposes (e.g., MRI, CT, dental procedures, minor surgeries, and endoscopic procedures). This introduces variability, as some procedures prioritise analgesia or anxiolysis over sleep induction, leading to inconsistent dosing protocols or outcome measures. Additionally, there is age-related variability. The participants’ ages ranged widely across the studies, complicating cross-study comparisons. Such variability in study design complicates the accurate characterization of dose–response relationships and safety profiles specific to electrophysiological auditory tests.

These limitations hinder the derivation of universal sedation guidelines for paediatric electrophysiological procedures. Future studies should prioritise age-stratified cohorts and standardised protocols to isolate sedation efficacy from analgesic/anxiolytic effects.

## 4. Conclusions

Electrophysiological testing in children requires the patient to be asleep to minimise the artifacts caused by muscle activity and movement. Natural sleep is ideal due to the absence of pharmacological side effects; however, it is often unpredictable and may not provide the necessary immobility for accurate testing, Therefore, pharmacologically induced sleep is frequently employed to ensure a calm and still patient, facilitating a more predictable and efficient testing process. Oral or intranasal sedation techniques allow for ABR and ASSR testing to be conducted outside the operating room, offering the advantage of discharging the patient home once they have fully awakened. Testing within the operating room should be reserved for cases where oral or intranasal sedation is contraindicated or has proven ineffective.

## Figures and Tables

**Table 1:** Summary table regarding the oral, intranasal, rectal, or intravenous routes, advantages, disadvantages, and indications.

	Advantages	Disadvantages	Indication
Oral	Non-invasive Easy to administer Minimal equipment required	Slow onset Less predictable absorption and effect Taste complains, vomiting Limited titration possibilities	Mild/moderate sedation
Intranasal	Rapid onset Non-invasive More rapid absorption than oral administration	Limited volume Potential nasal irritation Taste complains	Mild/moderate sedation
Rectal	More rapid onset than oral intake Useful for children who refuse oral or nasal routes	Variable absorption Discomfort and embarrassment Less precise titration	Mild/moderate sedation
Intravenous	Fastest onset Precise control over sedation depth Easy to titrate and reverse	Invasive Requires venous access and monitoring More resources and trained personnel	Rapid, deep sedation Prolonged interventions Precisely titratable sedation

**Table 2:** A comprehensive overview of the medications used in paediatric sedation.

Substance	Route	Dose	Onset	Duration	Efficacy	Side Effects
Melatonin	Oral	5 mg + additional dose in 48.5% of cases [32] 0.25–20 mg [29]	15–55 min [32]	15–55 min; average of 30.39 min [32]	65–86.7% [29]	Nothing significant
Chloral hydrate	Oral	50 mg/kg in children < 2 y 75 mg/kg in children >2 y Maximum single dose of 1000 mg [36]; 30–100 mg/kg [38]	10–75 min [37]		90% [38]–95.9% [36]	Respiratory depression, apnoea, hypoxemia, vomiting, bradycardia, prolonged sedation, paradoxical reaction, cardiovascular depression, and prolonged sleep (>2 h) [40,41]
	Rectal	20–80 mg/kg [40]	16.41 min [41]	71.59 ± 20.60 min [41]	97% [41]	
Triclofos [43]	Oral	50 mg/kg	30 min	90 min	93.1%	Dizziness, irritability, and vomiting
Hydroxyzine dihydrochloride	Oral	2 mg/kg [38]	15–60 min [44]	4–6 h [44]		Prolonged QT/QTc intervals, hyper-sedation, seizures, stupor, nausea, and vomiting [42,45]
Midazolam	Oral	0.32 ± 0.10 mg/kg [45] 0.5 mg/kg [54]	10–15 min [57]			Paradoxical reactions, nausea, vomiting, and respiratory events at doses over 0.5 mg/kg [55]
	Rectal	0.25–0.5 mg/kg				
	Intranasal [58]	0.5 mg/kg	5–10 min	108 min	51%	
	Intravenous	2–2.5 mg/kg + 1 mg every 2–3 min [83]	2–3 min [83]			
	Intramuscular	0.05–0.15 mg/kg, maximum of 10 mg [54]	10–20 min [54]			
Dexmedetomidine	Intranasal	2–4 µg/kg [66]	10–60 min; average of 22 min [62]		89% [64] 82.5 [66]–100% [62] 96.6% [67]	Hypotension, bradycardia, and oxygen desaturation [68,69,70]
	Intravenous [92]	1 µg/kg	3–5 min	15 min		
Dexmedetomidine with midazolam	Intranasal DEX + oral midazolam	3 µg/kg DEX + 0.1–0.2 mg/kg midazolam in syrup [66]	12–20 min; average of 15 min [66]	60–91.3 min; average of 73 min [66]	97.5% [66]	Hypotension and bradycardia [66]
Pentobarbital	Intrarectal pentobarbital ± alimemazine [74]	60 mg/12 kg, 75 mg/15 kg, and 90 mg/18 kg	10–60 min; mean of 64 ± 40 min		89.8%	Hypotension, respiratory disturbances, prolonged recovery time, and paradoxical reactions [72]
	Oral	4–8 mg/kg [72]			82% [73]	
Clonidine	Oral	4 µg/kg	10–60 min; mean of 45 min		88%	Drowsiness, dry mouth, bradycardia, and orthostatic hypotension [79]
	Rectal with atropine [83]	2.5–5 µg/kg				
	Intramuscular [83]	2 µg/kg				
	Intravenous with midazolam [83]	1–2 µg/kg/h + 50 µg/kg/h midazolam				
Ketamine	Intravenous	1–3 µg/kg [84]				Nausea, vomiting, hypersalivation, dizziness, diplopia, drowsiness, dysphoria, confusion, and hallucinations [86]
	Intramuscular	5–10 µg/kg [84]	1 min [84]	15–30 min [84]		
Propofol	Intravenous	2–3 mg/kg [88] Bolus at 0.8 mg/kg + continuous infusion at 0.1 mg/kg/min [91]	15–30 s [89]	1–3 min [88]		Apnoea, desaturation, bradycardia, and hypotension [90]
Ketamine with propofol [99]	Intravenous	1.5 mg/kg propofol + 0.5 mg/kg ketamine				Fewer side effects than propofol alone; nausea and vomiting
Ketamine with midazolam [100]	Intravenous	Ketamine 1 mg/kg + midazolam 0.1 mg/kg	30 s	15–60 min		
Propofol with dexmedetomidine [101]	Intravenous	0.8 µg/mL propofol + 0.5 µg/kg dexmedetomidine + 0.2 µg/kg/h dexmedetomidine				
Nitrous oxide [93]	Inhalation	5–6 L/min [93]	2–4 min	3–5 min after discontinuation		Nausea, vomiting, dizziness, and confusion [95]
Fentanyl	Intravenous	1–1.5 µg/kg + 1 µg/kg every 3 min [82]	1–2 min	30–60 min [82]		Hypotension, bradycardia, and respiratory depression

**Table 3:** Sedation strategies based on age and special conditions.

	<6 Months	6–12 Months	1–5 Years	>5 Years, Uncooperative, and Special Needs/ASA III–V
Natural sleep	Preferred	When is possible		When is possible
Melatonin	0.25 mg [29]	5–20 mg [29,32]		
Chloral hydrate	Orally 40–50 mg/kg [35,65] Rectally 20–80 mg/kg [40]	40 mg/kg ± 40 mg/kg [39]		
Dexmedetomidine	Intranasally 1–3 µg/kg [41]			
Dexmedetomidine + midazolam	3 µg/kg + 0.1–0.2 mg/kg midazolam [66]			Possible for uncooperative children
Deep sedation i.v. or i.m./general anaesthesia	Conducted by anaesthesiologists			Indicated for children with complex conditions

## Abbreviations

The following abbreviations are used in this manuscript:

ABR	Auditory brainstem response
ASSR	Auditory steady-state response
DEX	Dexmedetomidine
ASA	American Society of Anesthesiologists
